# Screening of 14 Lactic Acid Bacteria for Fermentative Isomalto/Malto-Polysaccharide Synthesis

**DOI:** 10.1021/acs.jafc.4c09286

**Published:** 2025-01-27

**Authors:** Nele Brand, Daniel Wefers

**Affiliations:** † Institute of Chemistry, Food Chemistry, Martin Luther University Halle-Wittenberg, Kurt-Mothes-Str. 2, 06120 Halle (Saale), Germany

**Keywords:** 4,6-α-glucanotransferases, maltodextrin, fermentation, sourdough, starch modification

## Abstract

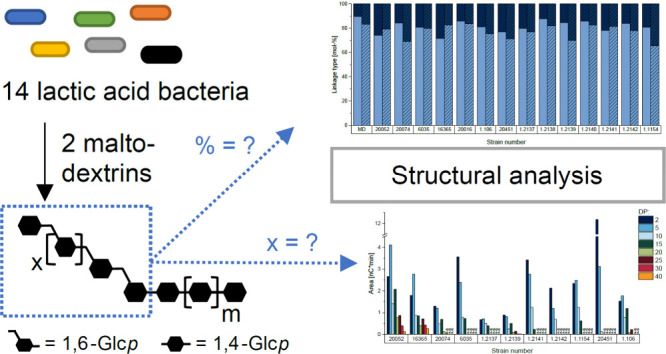

Some lactic acid bacteria such as *Limosilactobacillus reuteri* or *Fructilactobacillus sanfranciscensis* contain genes encoding 4,6-α-glucanotransferases. These enzymes convert starch and maltodextrins into isomalto/malto-polysaccharides (IMMPs). Many studies focused on the properties of recombinant glucanotransferases, but limited knowledge is available on fermentative synthesis. However, this aspect would be important for the *in situ* IMMP formation in fermented foods such as sourdough. Therefore, the aim of this study was to investigate the IMMP synthesis of 14 different lactic acid bacteria. We demonstrated that 11 of the investigated strains formed IMMPs with varying structural compositions from different maltodextrins. The portions of α-1,6-linkages depended on the bacterial strain and composition of the maltodextrin. By using different analytical approaches, it was demonstrated that linear chains of α-1,6-linked glucopyranoses with varying lengths were formed. Thus, the 11 IMMP-producing strains have high potential for an *in situ* synthesis of IMMPs in fermented foods such as sourdough.

## 1 Introduction

Starch is one of the main carbohydrate sources in the human diet and is composed of varying amounts of amylose and amylopectin. However, starch is mostly digestible in the small intestine; therefore, the consumption of food products rich in starch can contribute to excessive energy intake. Consequently, there is increasing interest in starch-modifying enzymes which convert starch into dietary fibers, prebiotics, or other functional polysaccharides. 4,6-α-glucanotransferases are starch-converting enzymes from the GH70 family which display the evolutionary intermediate between the GH13 and GH70 families.^1^ Three subfamilies of these enzymes are known: GtfB, GtfC, and GtfD.^2,3^ Most of the already described 4,6-α-glucanotransferases belong to the GtfB subfamily and are mainly found in different lactic acid bacteria. Almost all GtfB enzymes cleave α-1,4-glucosidic linkages and subsequently form α-1,6-linkages.^4,5^ This results in reaction products that are referred to as isomalto/malto-polysaccharides (IMMPs). IMMPs contain an α-1,4-linked chain on the reducing end and an α-1,6-linked chain at the nonreducing end. By use of optimized conditions, IMMPs with up to 92% α-1,6-linkages can be formed. Several studies indicated that IMMPs act as dietary fiber and have prebiotic properties.^6−8^

GtfB-like 4,6-α-glucanotransferases are further divided into two types: GtfB type I enzymes form linear chains of α-1,6-linked glucopyranoses, while GtfB type II enzymes are able to form branched, reuteran-like products.^9^ Several GtfB enzymes were described, many of them from *Limosilactobacillus* (*Llb*.) strains.^1,9−14^ Furthermore, GtfB enzymes were also found in other genera of lactic acid bacteria such as *Fructilactobacillus* (*Flb*.), *Weissella*, and *Streptococcus*.^15−19^ The products of the individual enzymes differ in the portion of 1,6-glucosidic linkages, which also depends on the substrate.^1,4,6,9−15,17−24^ The lengths of the 1,6-linked chains in IMMPs differ as well, but there are only a few studies in which this parameter was analyzed in detail.^25−27^

Previous research almost exclusively focused on the characterization and engineering of recombinant 4,6-α-glucanotransferases, whereas the fermentative synthesis of IMMPs by lactic acid bacteria has rarely been investigated. Bai et al. fermentatively synthesized IMMPs from different maltodextrins and starches by using several *Llb. reuteri* strains, however, only the IMMPs from *Llb. reuteri* 121 were analyzed in detail.^28^ Nevertheless, all strains were able to form 1,6-linkages from maltodextrins. Furthermore, Bai et al. showed that 4,6-α-glucanotransferases are cell-associated.^28^ However, the production of IMMPs by different lactic acid bacteria and the detailed analysis of the fermentatively formed reaction products remain largely unexplored. Investigating the fermentative synthesis of IMMPs is of great importance because the corresponding strains could be used as starter cultures for starch-containing fermented foods, such as sourdough.

Therefore, the aim of this study was to screen different lactic acid bacteria for their ability to synthesize IMMPs from maltodextrins and to gain deeper insight into the structure of the synthesized IMMPs. For this purpose, 14 lactic acid bacteria that were mostly isolated from sourdough or other starch-containing environments were investigated.

## 2 Materials and Methods

### 2.1 Materials

*Llb. reuteri* DSM 20016, *Flb. sanfranciscensis* DSM 20451, *Llb. panis* DSM 6035, *Llb. fermentum* DSM 20052, *Lactobacillus* (*Lb*.) *delbrueckii* subsp. *delbrueckii* DSM 20074, and *Lactiplantibacillus* (*Lpb*.) *argentoratensis* DSM 16365 were purchased from the German Collection of Microorganisms and Cell Cultures (DSMZ) GmbH, Braunschweig, Germany. *Llb. reuteri* TMW 1.106, *Flb. sanfranciscensis* TMW 1.1154, *Flb. sanfranciscensis* TMW 1.2137, *Flb. sanfranciscensis* TMW 1.2138, *Flb. sanfranciscensis* TMW 1.2139, *Flb. sanfranciscensis* TMW 1.2140, *Flb. sanfranciscensis* TMW 1.2141, and *Flb. sanfranciscensis* TMW 1.2142 were kindly provided by Prof. Rudi Vogel and Prof. Fabio Minervini. If not stated otherwise, all chemicals used were of “p.a.” grade or better and were purchased from Carl Roth (Karlsruhe, Germany), Merck (Darmstadt, Germany), Thermo Fisher Scientific (Waltham, MA USA), VWR (Darmstadt, Germany), and Grüssing GmbH (Filsum, Germany). Two maltodextrins with dextrose equivalents (DE) of 6.6 (MD6.6) and 16.8 (MD16.8) were purchased from Merck (Darmstadt, Germany). *Endo*-Dextranase from *Chaetomium sp*. (EC 3.2.1.11, 8000 U/mL), isopullulanase from *Aspergillus niger* (EC 3.2.1.57, 1000 U/mL), isoamylase HP from *Pseudomonas sp.* (EC 3.2.1.68, 500 U/mL), and β-amylase from barley (EC 3.2.1.2, 10 000 U/mL) were purchased from Megazyme (Bray, Ireland).

### 2.2 Synthesis and Isolation of IMMPs

Cultivation of the above-mentioned strains was carried out in modified Spicher medium^29^ (pH 5.4), which was composed of 10 g/L peptone from casein, 2 g/L meat extract, 7 g/L yeast extract, 2 g/L sodium gluconate, 5 g/L sodium acetate trihydrate, 5 g/L diammonium hydrogen citrate, 2.5 g/L potassium dihydrogen phosphate, 0.5 g/L cystein hydrochloride, 1 g/L tween 80, 0.2 mg/L of biotin, folic acid, nicotinic acid, pyridoxal phosphate, thiamine, riboflavin, cobalamin and pantothenic acid, 2 mL/L Spicher salt mix (5 g/L magnesium sulfate heptahydrate, 1.88 g/L manganese sulfate tetrahydrate, 1.25 g/L iron sulfate heptahydrate in water), 7 g/L fructose, 7 g/L glucose, and 7 g/L maltose. First, a preculture was grown statically at 30 °C for 1–7 days until an OD_600_ of at least 0.5 was reached. For IMMP production, 25 mL of preculture were added to 280 mL of MD-Spicher medium (modified Spicher medium with 5% (*m*/*m*) maltodextrin as sole carbohydrate source) in a 250 mL bottle and incubated at 30 °C for 4 days. After incubation, the cells were removed by centrifugation at 10 070 rcf for 15 min. Subsequently, the IMMPs were precipitated from the supernatant by adding 4 volumes of ethanol, incubated overnight, and isolated by centrifugation at 4536 rcf and 10 °C for 30 min. The precipitate was redissolved, and the precipitation step was repeated to achieve a higher purity. Subsequently, the isolated IMMPs were redissolved in ultrapure water and lyophilized. For comparison, the unfermented maltodextrins were isolated from the medium under the same conditions and used as a control in further experiments.

### 2.3 ^1^H NMR Spectroscopy

Lyophilized IMMPs were dissolved in D_2_O (15 mg/mL) and acetone was added as a reference (referenced to 2.22 ppm according to Gottlieb et al.^30^). Samples were analyzed on a 400 MHz VNMRS or a 500 MHz DD2 spectrometer (Agilent, Santa Clara, CA, USA).

### 2.4 Methylation Analysis

Methylation analysis of the IMMPs obtained from MD16.8 was performed as described by Ernst et al.^31^ First, 1 mg of each sample was swollen in 2 mL of DMSO overnight, and then about 100 mg of NaOH_(s)_ was freshly ground under an argon atmosphere and added to the sample. After incubation in an ultrasonic bath for 90 min, the samples were left at room temperature for another 90 min. For methylation, 1 mL of methyl iodide was added and methylation was carried out at room temperature for 1 h. Subsequently, 3 mL of dichloromethane were added and extracted with 5 mL of 0.1 M aqueous sodium thiosulfate solution. The organic phase was washed twice with 5 mL of ultrapure water. Subsequently, dichloromethane was evaporated and the samples were dried in a vacuum oven at 40 °C overnight. To ensure complete methylation, the procedure was repeated. After methylation, the samples were hydrolyzed into partially methylated monosaccharides with 2 mL of 2 M aqueous trifluoroacetic acid at 121 °C for 90 min. Trifluoroacetic acid was removed by evaporation. Reduction was carried out with 20 mg of sodium borodeuteride in 0.3 mL of 2 M aqueous NH_3_ at room temperature for 1 h. To terminate the reaction, 100 μL of glacial acetic acid was added to each sample. For acetylation, 450 μL of 1-methyl imidazole and 3 mL of acetic anhydride were added under ice cooling and the reaction was carried out at room temperature for 30 min. To remove the excess acetic anhydride, 3 mL of ultrapure water were added under ice cooling. The solution was extracted with 5 mL of dichloromethane and the organic phase was washed three times with ultrapure water. Residual water was frozen out at −18 °C overnight. The partially methylated alditol acetateswere separated by GC-MS and identified by their mass spectra. Quantification was carried out by GC-FID and using the response factors by Sweet et al.^32^ Detailed conditions were described by Ernst et al.^31^

### 2.5 Fingerprint Analysis

Fingerprint analysis of the IMMPs was performed as described by van der Zaal et al.^25^ with minor modifications. IMMPs were dissolved in sodium acetate buffer (20 mM NaOAc with 5 mM CaCl_2_, pH 5.5; IMMP concentration, 2.5 mg/mL). Subsequently, 0.8 U isoamylase HP, 2 U β-amylase, and 0.8 U isopullulanase were added to 1 mL of sample solution, and the mixture was incubated at 40 °C for 4 h. To terminate the reaction, enzymes were inactivated at 95 °C for 10 min and removed by centrifugation (4 °C, 21 750 rcf, 20 min). Hydrolyzed samples were diluted and analyzed by high-performance anion exchange chromatography with pulsed amperometric detection (HPAEC-PAD) on an ICS-6000 system (Thermo Fisher Scientific, Waltham, MA, USA) equipped with a Carbo-Pac PA200 column (250 mm × 3 mm inner diameter, 5.5 μm particle size, Thermo Fisher Scientific). The column temperature was 30 °C and the detector temperature was 25 °C. The following gradient was used with a flow rate of 0.4 mL/min: Column equilibration for 20 min with 10 mM NaOH; 0–10 min, isocratic with 10 mM NaOH; 10–20 min, linear gradient from 10 mM NaOH to 105 mM NaOH; 20–85 min, linear gradient from 105 mM NaOH to 105 mM NaOH + 200 mM NaOAc; 85–95 min, linear gradient from 105 mM NaOH + 250 mM NaOAc to 200 mM NaOH + 500 mM NaOAc; 95–110 min, isocratic with 200 mM NaOH + 500 mM NaOAc; 110–125 min, isocratic with 200 mM NaOH. The unfermented maltodextrins isolated from the medium were analyzed under the same conditions and used as control. To identify the linear α-1,6-linked isomalto-oligosaccharides in the chromatogram, the linear dextran from *Ligilactobacillus animalis* TMW 1.971^33,34^ was partially hydrolyzed with trifluoroacetic acid and used as a reference mixture.

## 3 Results and Discussion

### 3.1 Fermentative Synthesis of IMMPs

As mentioned above, the 14 lactic acid bacteria used in this study were selected based on the source of isolation, which is shown in Table 1. Furthermore, a gene encoding a 4,6-α-glucanotransferase was found in all strains except *Flb. sanfranciscensis* TMW 1.2138.

**Table 1:** Origin of the Lactic Acid Bacteria Used in This Study

bacterial strain	descriptor	origin
*Fructilactobacillus sanfranciscensis* TMW 1.1154	1.1154	sourdough^35^
*Fructilactobacillus sanfranciscensis* TMW 1.2137	1.2137	sourdough^35^
*Fructilactobacillus sanfranciscensis* TMW 1.2138	1.2138	sourdough^35^
*Fructilactobacillus sanfranciscensis* TMW 1.2139	1.2139	sourdough^35^
*Fructilactobacillus sanfranciscensis* TMW 1.2140	1.2140	sourdough^35^
*Fructilactobacillus sanfranciscensis* TMW 1.2141	1.2141	sourdough^35^
*Fructilactobacillus sanfranciscensis* TMW 1.2142	1.2142	sourdough^35^
*Fructilactobacillus sanfranciscensis* DSM 20451	20451	sourdough^35^
*Limosilactobacillus reuteri* TMW 1.106	1.106	sourdough^36^
*Limosilactobacillus reuteri* DSM 20016	20016	human feces^37^
*Limosilactobacillus fermentum* DSM 20052	20052	fermented beets^38^
*Limosilactobacillus panis* DSM 6035	6035	type II sourdough^39^
*Lactobacillus delbrueckii* subsp. *delbrueckii* DSM 20074	20074	sour grain mash^40^
*Lactiplantibacillus argentoratensis* DSM 16365	16365	fermented cassava roots^41^

To obtain insights into the effect of the substrate size on the fermentative IMMP formation, two maltodextrins with different dextrose equivalents (6.6 and 16.8) were used. The HPAEC chromatograms of the two maltodextrins clearly demonstrated that they contain a broad range of malto-oligosaccharides and starch fragments (Figure S1 and S2). As expected, MD6.6 contained clearly higher portions of high molecular weight compounds, whereas MD16.8 was composed of rather small starch fragments. Fermentation was carried out for 4 days to ensure sufficient bacterial growth and to provide enough time for enzymatic reactions. Despite some variation, the amounts of precipitated polysaccharides were comparable before and after fermentation for each maltodextrin (Figure S3). About 81% were precipitated from unfermented MD6.6, whereas the portion of precipitated polysaccharides mostly ranged from 60% to 85% after fermentation. For the untreated MD16.8, the polysaccharide yield was about 42%, whereas between 30 and 45% of the maltodextrin were recovered after fermentation. Thus, the reactions during fermentation did not result in a substantial decrease in the amount of precipitable compounds. The yield of polysaccharides precipitated with a fixed volume ratio of ethanol and water largely depends on the size of the glucans.^42^ With four volumes of ethanol, oligo- and polysaccharides with a molecular weight up to approximately 3 kDa may be not precipitated and remain in solution.^42^ This explains the significantly lower amount of precipitable material in MD16.8. Bai et al. used a maltodextrin with a dextrose equivalent of 13.0–17.0 for fermentative synthesis and precipitated the IMMPs with two volumes of cold ethanol. The authors stated that low yields were achieved, but the exact amounts were not published.^28^ It was suggested that the low yields were caused by a high hydrolytic activity of the respective 4,6-α-glucanotransferases,^28^ but the comparably high portion of low molecular weight compounds in the maltodextrins used as well as the lower portion of ethanol may also be an explanation. However, in our case, the yields of precipitable polysaccharides suggest that the hydrolytic activity is at least not high enough to largely degrade polymeric maltodextrin. The varying yields may be a result of different hydrolytic activities, but as this parameter was not determined, it is not possible to draw unambiguous conclusions.

### 3.2 Structural Composition of the Fermentation Products

**Figure 1:**
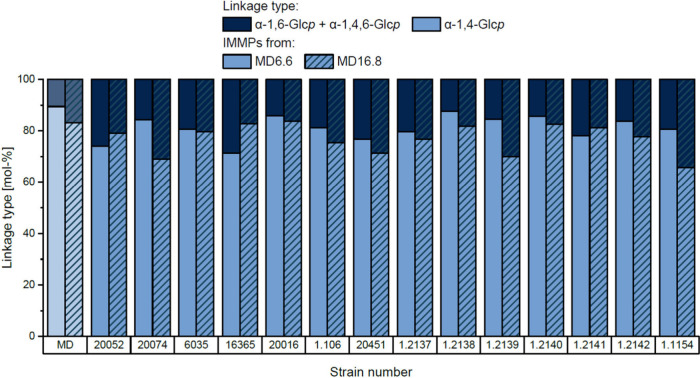
Portions of α-1,6-/1,4,6-linked glucopyranose units (α-1,4,6-/1,6-Glc*p*, dark blue) and α-1,4-linked glucopyranose units (α-1,4-Glc*p*, light blue) in the two maltodextrins (MD) and the products resulting from the fermentation with 14 different lactic acid bacteria strains at 30 °C. The strains corresponding to the strain numbers are shown in Table 1. MD6.6 = maltodextrin with a dextrose equivalent of 6.6, MD16.8 = maltodextrin with a dextrose equivalent of 16.8.

To get an overview of the portions of α-1,6-linked glucopyranoses after fermentation, all reaction products and the maltodextrins were analyzed by ^1^H NMR spectroscopy. To estimate the portions of 1,4- and 1,6-linked glucopyranose units, the signals of their anomeric protons were integrated (Figures S4, S5 and S6). However, it has to be considered that the maltodextrins contain some branched oligosaccharides and polysaccharides and that the side chains give the same signal as 1,6-linked units in IMMPs. Therefore, the portions of these natively present structural elements are included in the portion of the 1,6-linked glucose units. The NMR spectroscopically determined portions of α-1,6- and α-1,4-linkages in the untreated maltodextrins, and the fermentation products are shown in Figure 1.

The fermentation products obtained from the fermentation of the two maltodextrins with *Llb. reuteri* DSM 20016, *Flb. sanfranciscensis* TMW 1.2138 and 1.2140 showed almost the same portions of α-1,6- and 1,4-linkages as the respective unfermented maltodextrins. Therefore, these strains seemed to be unable to synthesize significant amounts of IMMPs under the conditions used. This was unexpected in the case of *Llb. reuteri* DSM 20016, because the recombinant, *N*-terminally truncated 4,6-α-glucanotransferase GtfW of *Llb. reuteri* DSM 20016, was already used to synthesize IMMPs from different malto-oligosaccharides, maltodextrin and amylose V.^13,20^ Moreover, Bai et al. used *Llb. reuteri* DSM 20016 to fermentatively produce IMMPs from maltodextrin with a dextrose equivalent of 13.0–17.0, although the yield was low.^28^ Differences in the fermentation conditions (especially the fermentation temperature) that influence the hydrolysis/transglycosylation ratio of the 4,6-α-glucanotransferases could be a reason for this discrepancy. Indeed, the ^1^H NMR spectra of the IMMPs from *Llb. reuteri* DSM 20016 indicate a hydrolytic activity of the strain, because the anomeric signals which correspond to glucopyranoses at the reducing end and free glucose units have a higher intensity than in the ^1^H NMR spectra of the respective maltodextrin and the other IMMPs (Figure S5 and S6). An increased hydrolytic activity could result in smaller IMMPs that are not precipitated. Therefore, optimization of the fermentation conditions could result in an increased portion of 1,6-linkages in the polymeric fraction.

For all other strains used, levels of α-1,6-linked glucopyranoses higher than those in the respective maltodextrin were found in at least one fermentation product. Therefore, several of the selected bacteria were able to produce IMMPs from maltodextrin under the conditions used. The highest portion of α-1,6-linked glucopyranoses (34.4%) was found in the IMMPs formed by *Flb. sanfranciscensis* TMW 1.1154 from MD16.8. These results align well with those previously reported by Bai et al., who fermented different maltodextrins with *Llb. reuteri* 121 and found 19–35% α-1,6-linkages in the fermentation products (11–52% were obtained by using different starches).^28^ In other studies, the modification of maltodextrins by recombinant 4,6-α-glucanotransferases also resulted in comparable portions of 1,6-linkages. For example, the modification of different maltodextrins with the recombinant GtfB from *Llb. reuteri* 121 yielded 24–36% 1,6-linkages.^6,20^ Furthermore, 32% 1,6-linkages were formed from maltodextrin with a dextrose equivalent of 13.0–17.0 by using the GtfW from *Llb. reuteri* DSM 20016,^20^ whereas the GtfB from *Flb. sanfranciscensis* TMW 1.1304 produced 35% 1,6-linkages from maltodextrin with a dextrose equivalent of 4.0–7.5.^17^ The slight variation between the portions of 1,6-linkages in the products synthesized by fermentation and recombinant enzymes could be a result of the cell association of the enzymes, different reaction conditions, or differences between the substrate–enzyme ratios.^28^ In addition, the metabolization of enzymatically released glucose or maltose units by the bacteria may limit the availability of acceptor molecules, which could also affect the product spectrum.^28^

Notably, the portion of α-1,6-linkages depended on the maltodextrin used. Some strains formed higher portions of α-1,6-linkages with MD6.6 compared to MD16.8 (e.g., *Lpb. argentoratensis* DSM 16365). In contrast, other strains such as *Lb. delbrueckii* subsp. *delbrueckii* DSM 20074 synthesized more 1,6-linkages from MD16.8. However, some strains produced IMMPs with comparable portions of 1,6-linkages from the two maltodextrins (e.g., *Llb. panis* DSM 6035). Differences in the substrate preferences of recombinant 4,6-α-glucanotransferases were already described in several studies,^10,13,43^ thus the observed differences could be explained by the varying properties of the respective 4,6-α-glucanotransferases.

Since some of the selected strains preferentially grow at higher temperatures, maltodextrin fermentation with *Llb. reuteri* TMW 1.106, *Llb. fermentum* DSM 20052, and *Lb. delbrueckii* subsp. *delbrueckii* DSM 20074 was also carried out at 37 °C. All three strains were able to produce IMMPs under these conditions as well, and the portions of 1,6-linkages in the IMMPs were roughly comparable (data not shown). To allow for better comparability with the other strains, we decided to present the data obtained from the fermentation at 30 °C throughout the manuscript.

**Figure 2:**
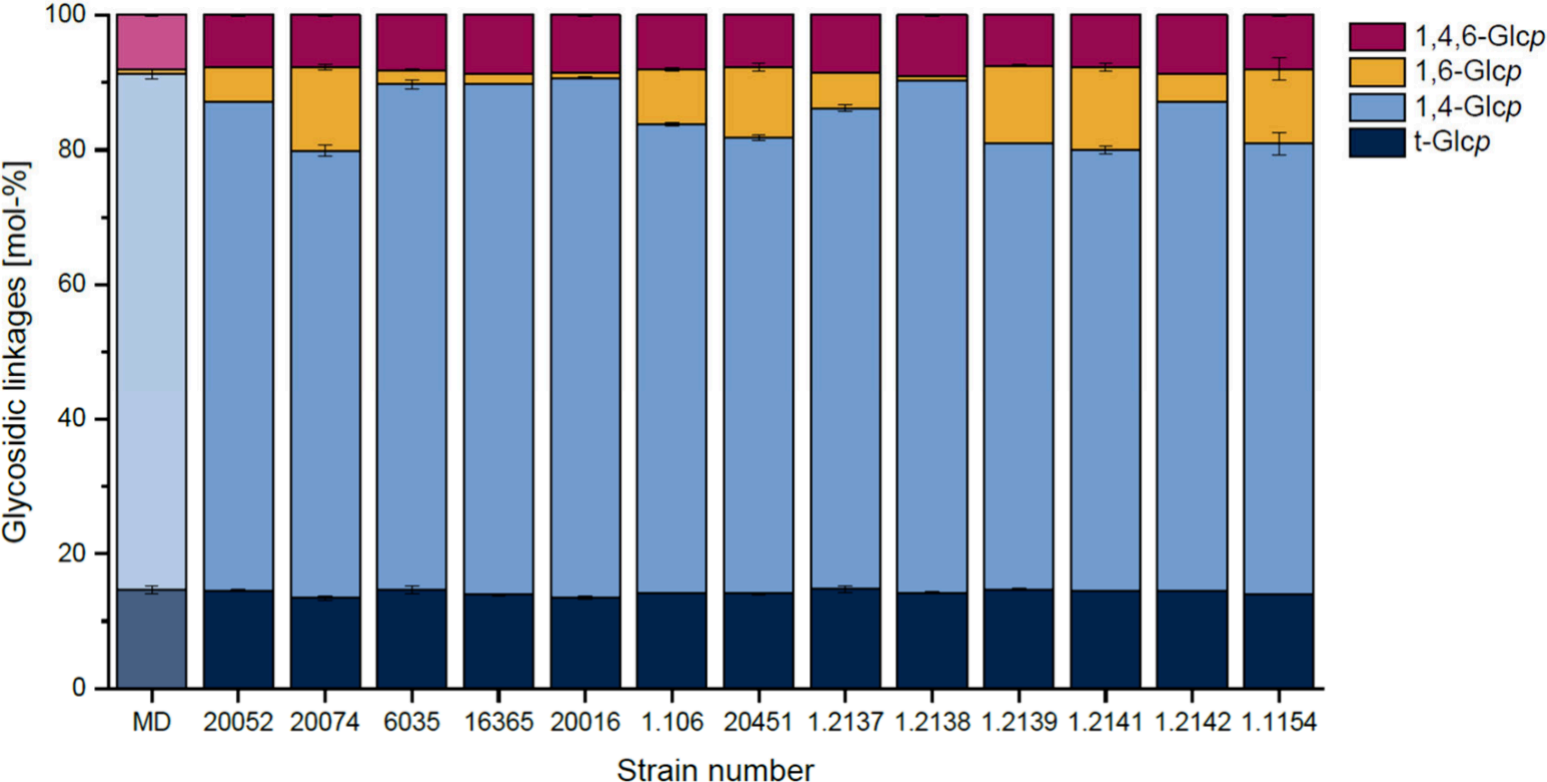
Glycosidic linkages in the maltodextrin with a dextrose equivalent of 16.8 and the products obtained from the fermentation of this maltodextrin with different lactic acid bacteria. The strains corresponding to the strain numbers are shown in Table 1.

To confirm the portions of 1,6-linkages obtained from ^1^H NMR spectroscopy and to obtain more information about the linkage types in the fermentation products, methylation analysis was carried out. Compared to ^1^H NMR spectroscopy, this method has the advantage that the portion of 1,6-glucopyranose units can be differentiated from the portion of branched, 1,4,6-linked glucopyranose units. This is relevant because several 4,6-α-glucanotransferases synthesize not only linear 1,6-linked chains, but also produce reuteran-like, branched glucan chains.^9,10,15,16^ In addition, methylation analysis can be used to confirm that some fermentation products do not contain more 1,6-linked glucopyranose units than untreated maltodextrin. Because it is not expected that the bacterial 4,6-α-glucanotransferases form different linkage types with different maltodextrins, only the fermentation products formed from MD16.8 were analyzed. Furthermore, only the fermentation products that showed increased portions of 1,6-linkages compared with maltodextrin were analyzed. However, the products from *Flb. sanfranciscensis* DSM 1.2138 were analyzed as control, and the products from *Llb. reuteri* DSM 20016 were analyzed because IMMP formation was expected due to previously described results.^28^ The results from the methylation analysis are shown in Figure 2.

Only four different linkage types were detected in every fermentation product: terminal glucose units as well as 1,4-linked, 1,4,6-linked, and 1,6-linked glucose units. Thus, as already expected from the results of ^1^H NMR spectroscopy, the formation of other linkage types can be excluded. Higher portions of 1,4,6-linked glucopyranose units in comparison to the unfermented maltodextrin were not observed, therefore, the results from methylation analysis suggest that the obtained IMMPs contained only unbranched 1,6-linked segments. The portions of 1,6-glucopyranose units in the fermentation products ranged between 1.6 and 12.2%. This is consistent with the results of ^1^H NMR spectroscopy considering that the NMR signal used for 1,6-linkages is also derived from ramifications of the maltodextrins and starch fragments (thus, 1,4,6-linked and in part terminal glucose units are detected as 1,6-linkages by NMR spectroscopy as shown in Figure S4). Nine strains contained clearly higher portions of 1,6-linkages than the maltodextrin, which unambiguously confirms IMMP formation. Although only comparably low portions of 1,6-linkages were detected for the polysaccharides from *Lpb. argentoratensis* DSM 16365, IMMP formation was clearly evident from the NMR results obtained for MD6.6. For the fermentation products from *Llb. panis* DSM 6035, the low portions of 1,6-linkages indicated IMMP formation, but to a quite low extent. As expected, the fermentation products of *Flb. sanfranciscensis* DSM 1.2138 did not show an increased portion of 1,6-linkages. Therefore, it was confirmed that the fermentation procedure did not alter the portion of 1,6-linkages due to other mechanisms. Notably, the methylation analysis results also confirmed that *Llb. reuteri* DSM 20016 did not seem to synthesize significant portions of 1,6-linkages. Altogether, 11 of the 14 selected lactic acid bacteria were able to synthesize α-1,6-linkages from at least one maltodextrin.

### 3.3 Size Distribution of the α-1,6-Linked Chains

To obtain a better understanding of the IMMP structures, the enzymatic fingerprinting method described by van der Zaal et al.^25^ was carried out. Because the length of the α-1,6-linked chains was of particular interest, the α-1,4-linked chains were enzymatically hydrolyzed by using isoamylase, β-amylase, and isopullulanase, and the liberated α-1,6-linked chains were analyzed by HPAEC-PAD. In addition, both maltodextrins were hydrolyzed and analyzed as control. To identify the liberated linear, α-1,6-linked oligo- and polysaccharides, the linear dextran from *Ligilactobacillus animalis* TMW 1.971 was partially hydrolyzed and used as a size standard.

**Figure 3:**
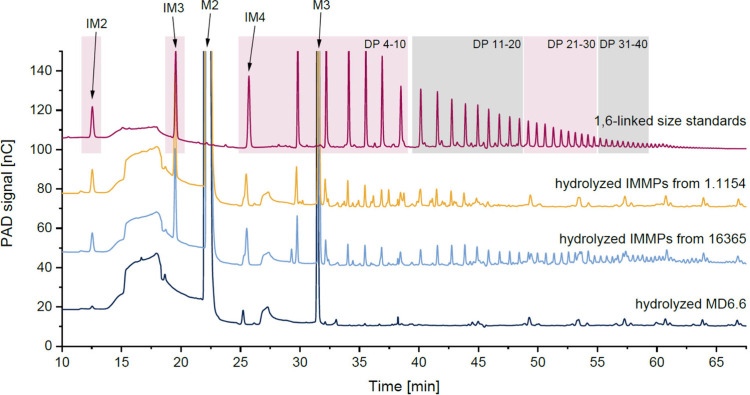
HPAEC-PAD chromatograms of the size standard (partially hydrolyzed linear dextran, red), the hydrolyzed isomalto/malto-polysaccharides (IMMPs) synthesized by *Fructilactobacillus sanfranciscensis* TMW 1.1154 (yellow) and *Lactiplantibacillus argentoratensis* DSM 16365 (light blue) from maltodextrin with a dextrose equivalent of 6.6, and the hydrolyzed maltodextrin with a dextrose equivalent of 6.6 (MD6.6, dark blue). The degrees of polymerization (DP) of the size standard peaks are marked with red and gray boxes. IM2 = isomaltose, IM3 = isomaltotriose, IM4 = isomaltotetraose, M2 = maltose, M3 = maltotriose.

Selected chromatograms of the hydrolysates are shown in Figure 3, while the chromatograms of all hydrolysates are compared in Figure S7 and Figure S8. By using the enzymatic profiling approach, it was possible to again demonstrate IMMP formation by the 11 strains through the detection of 1,6-linked oligo- and polysaccharides that are not present in the untreated maltodextrin. The absence of these products in the hydrolysates of the maltodextrins fermented by *Llb. reuteri* DSM 20016, *Flb. sanfranciscensis* TMW 1.2138, and *Flb. sanfranciscensis* TMW 1.2140 confirmed that these strains did not form IMMPs. Notably, clearly different peak patterns were observed in the chromatograms of the analyzed IMMPs. For most of the samples, the sizes of the liberated, 1,6-linked oligo- and polysaccharides showed significant variation. For example, larger 1,6-linked compounds were observed in the hydrolysate of the IMMPs from *Lpb. argentoratensis* DSM 16365, whereas the compounds present in the IMMPs from *Flb. sanfranciscensis* TMW 1.1154 were clearly smaller (Figure 3). Some chromatograms also showed additional peaks besides the ones derived from the 1,6-linked standards, for example, the chromatograms of the hydrolyzed IMMPs of the *Flb. sanfranciscensis* strains. Thus, some additional oligosaccharides were liberated from these fermentation products, indicating that the α-1,6-linked chains in the corresponding IMMPs contain other glycosidic linkages. For example, the IMMPs from the *Flb. sanfranciscensis* strains could contain 1,4-linked glucose units within the 1,6-linked chains, especially because of the linkage composition: all IMMPs showed similar portions of 1,4,6-linked glucose units but varying portions of 1,4- and 1,6-linked glucose units. Furthermore, additional glycosidic linkages were not detected.

**Figure 4:**
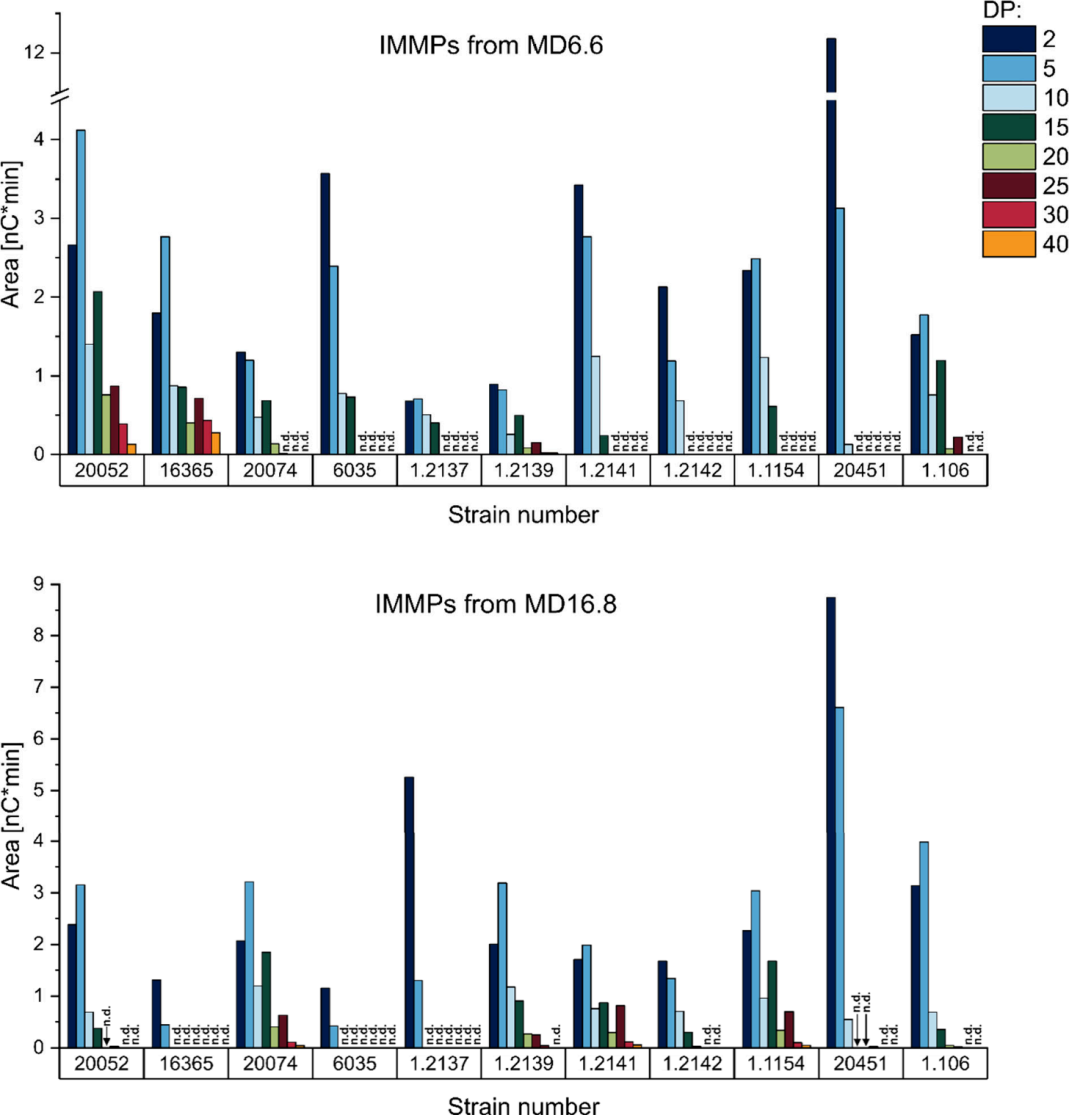
Peak areas of selected 1,6-linked oligo- and polysaccharides which were liberated by enzymatic hydrolysis from different isomalto/malto-polysaccharides (IMMPs). The IMMPs were synthesized by different lactic acid bacteria from maltodextrins with a dextrose equivalent of 6.6 (MD6.6) and 16.8 (MD16.8). The strains corresponding to the strain numbers are shown in Table 1. n.d. = not detected, DP = degree of polymerization.

As described above, the chromatograms of the enzymatically liberated α-1,6-linked chains contained linear oligo- and polysaccharides with different degrees of polymerization. To visualize the size distributions, the peaks representing selected oligo- and polysaccharides were integrated for each sample, and the obtained areas were used for comparison. However, it is important to note that this approach cannot give quantitative information because the detector response of these compounds is dependent on the degree of polymerization. An overview of the size distributions of the 1,6-linked chains in the IMMPs from MD6.6 and MD16.8 is shown in Figure 4.

The comparison of the abundance of the selected compounds demonstrated clear differences between the fermentatively synthesized IMMPs. For example, the IMMPs from *Flb. sanfranciscensis* DSM 20451 showed very short α-1,6-linked chains (dominated by DP 2 and 5) regardless of the maltodextrin which was used for fermentation. In contrast, the α-1,6-linked chains of the IMMPs from *Llb. fermentum* DSM 20052 and *Lpb. argentoratensis* DSM 16365 from MD6.6 were clearly longer (DPs up to >40), whereas the same strains produced only small 1,6-linked chains from MD16.8. Notably, other strains such as *Lb. delbrueckii* subsp. *delbrueckii* DSM 20074 and *Flb. sanfranciscensis* TMW 1.2139, 1.2141, and 1.1154 synthesized long α-1,6-linked chains from MD16.8. Therefore, not only the portion of 1,6-linkages, but also the length of the 1,6-linked chains depended on the strain as well as the substrate. Notably, the portion of α-1,6-glucopyranose units in the IMMPs and the length of the α-1,6-linked chains correlated in some cases, for example, for the IMMPs formed by *Llb. fermentum* DSM 20052 and *Lpb. argentoratensis* DSM 16365 from MD6.6. However, for other IMMPs, a higher abundance of 1,6-linkages was associated with the formation of shorter α-1,6-linked chains, e.g., for the IMMPs formed by *Llb. reuteri* TMW 1.106 and *Flb. sanfranciscensis* TMW 1.2137 from MD16.8. Therefore, the chain length is clearly dependent on the enzyme as well as the substrate.

Altogether, we clearly demonstrated that 11 different lactic acid bacteria were able to synthesize IMMPs from different maltodextrins. The obtained IMMPs differed in terms of the portion of the α-1,6-linkages and the size distribution of the α-1,6-linked chains. The structure of the IMMPs depended on the strain, as well as the maltodextrin used for the fermentation. It is likely that the obtained IMMP yields can be improved by adjusting the substrate or fermentation conditions. For example, the fermentation temperature, fermentation time, or portion of the substrate in the medium may influence the (hydrolytic) activity of the microbial 4,6-α-glucanotransferases and thus the IMMP yield. In addition, the structure of the IMMPs could be tailored using a certain combination of strain and substrate. Therefore, the investigated lactic acid bacteria have a high potential for application as starter cultures in fermented, starch-rich foods such as sourdough.

## Supporting Information Available

HPAEC-PAD chromatograms of the maltodextrins, yield of the fermentation products, assignment of the anomeric signals in the ^1^H NMR spectrum of IMMPs, ^1^H NMR spectra of the IMMPs, HPAEC-PAD chromatograms of the IMMPs after enzymatic hydrolysis (PDF)

## Abbreviations

IMMPs: isomalto/malto-polysaccharides
MD: maltodextrin
DE: dextrose equivalent
HPAEC-PAD: high-performance anion exchange chromatography with pulsed amperometric detection
Glc*p*: glucopyranose
*Llb.*: *Limosilactobacillus*
*Flb.*: *Fructilactobacillus*
*Lb.*: *Lactobacillus*
*Lpb.*: *Lactiplantibacillus*
