# Supplementary material for: Screening of 14 Lactic Acid Bacteria for Fermentative Isomalto/Malto-Polysaccharide Synthesis
Source: J Agric Food Chem. 2025 Jan 27;73(5):2970–7. doi: 10.1021/acs.jafc.4c09286 (PMC11803698; doi:10.1021/acs.jafc.4c09286)
Supplement: Supplementary file 1 — jf4c09286_si_001.pdf [file jf4c09286_si_001.pdf]

Supporting information

Screening of 14 lactic acid bacteria for fermentative isomalto/malto-polysaccharide synthesis

Nele Brand[1], Daniel Wefers[1]*

[1] Institute of Chemistry, Food Chemistry, Martin Luther University Halle-Wittenberg, 06120 Halle (Saale), Germany

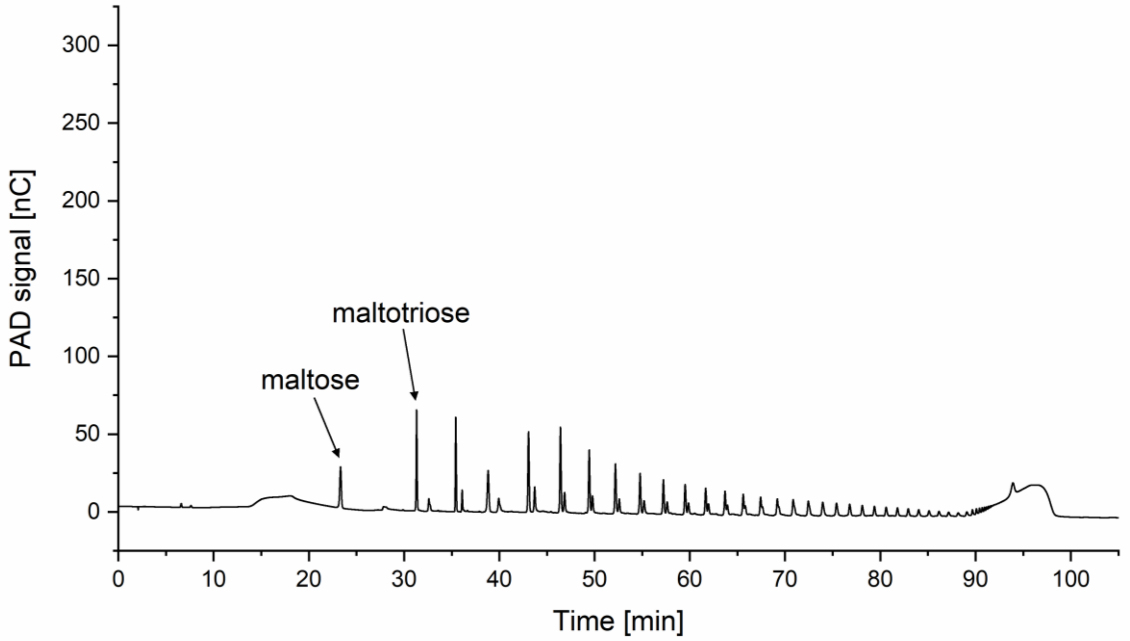

Figure S1: HPAEC-PAD chromatogram of the maltodextrin with a dextrose equivalent of 6.6.

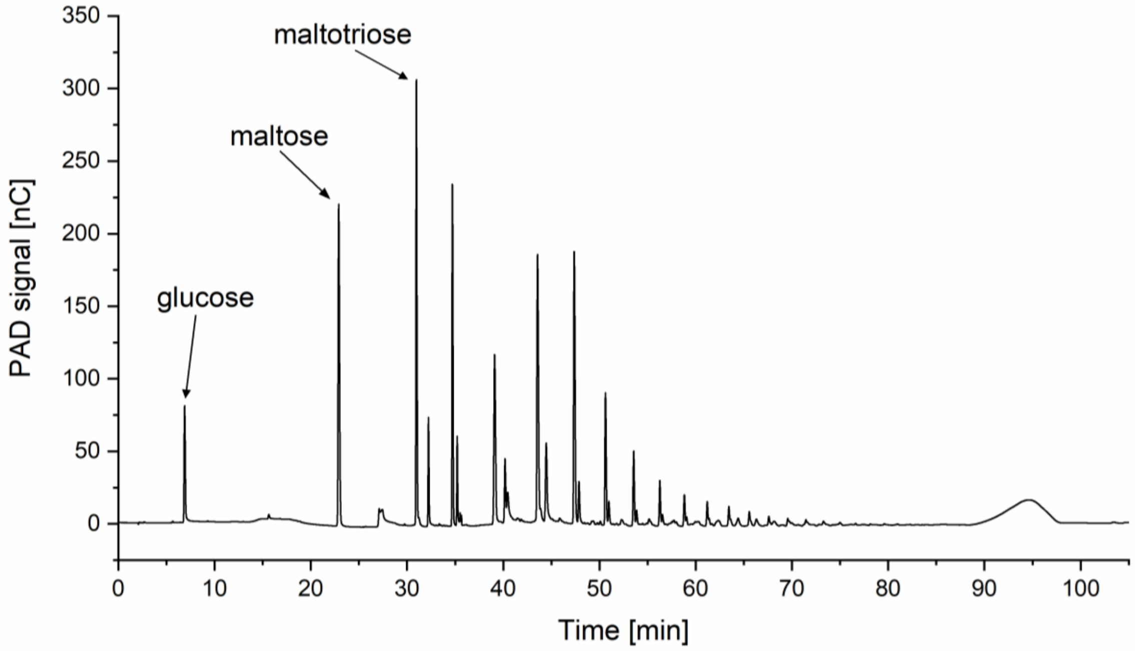

Figure S2: HPAEC-PAD chromatogram of the maltodextrin with a dextrose equivalent of 16.8.

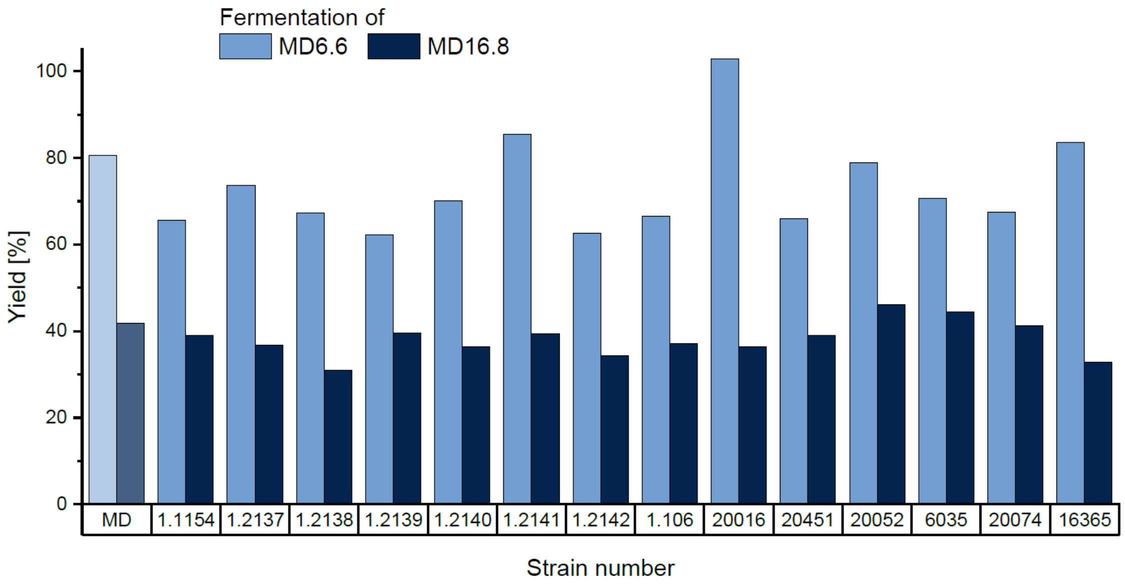

Figure S3: Polysaccharide yields obtained before (MD) and after fermentation of maltodextrins with a dextrose equivalent of 6.6 (MD6.6) and 16.8 (MD16.8) with 14 different lactic acid bacteria. The relative yield was calculated based on the amount of the maltodextrin that was added to the medium. The strains corresponding to the strain numbers are shown in Table 1.

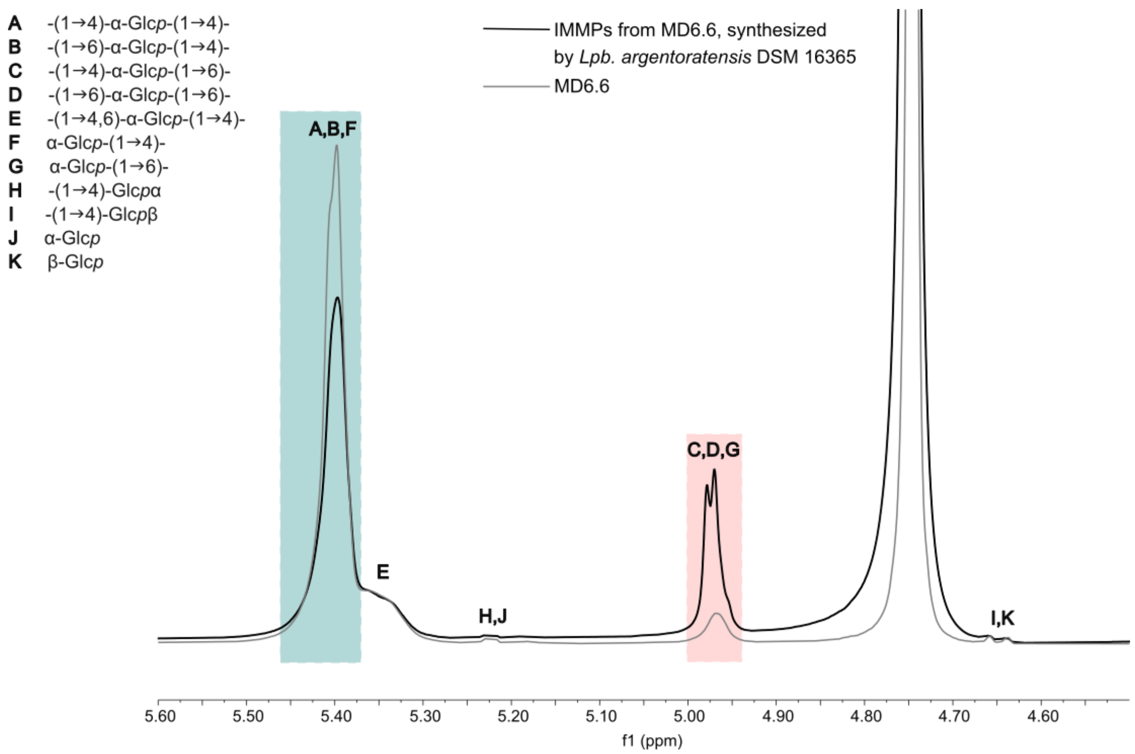

Figure S4. Assignment of the anomeric signals in the ^{1}H NMR spectra of maltodextrin with a dextrose equivalent of 6.6 (MD6.6, grey) and the isomalto/malto-polysaccharides (IMMPs) from MD6.6, synthesized by *Lactiplantibacillus* (*Lpb.*) *argentoratensis* DSM 16365 (black).

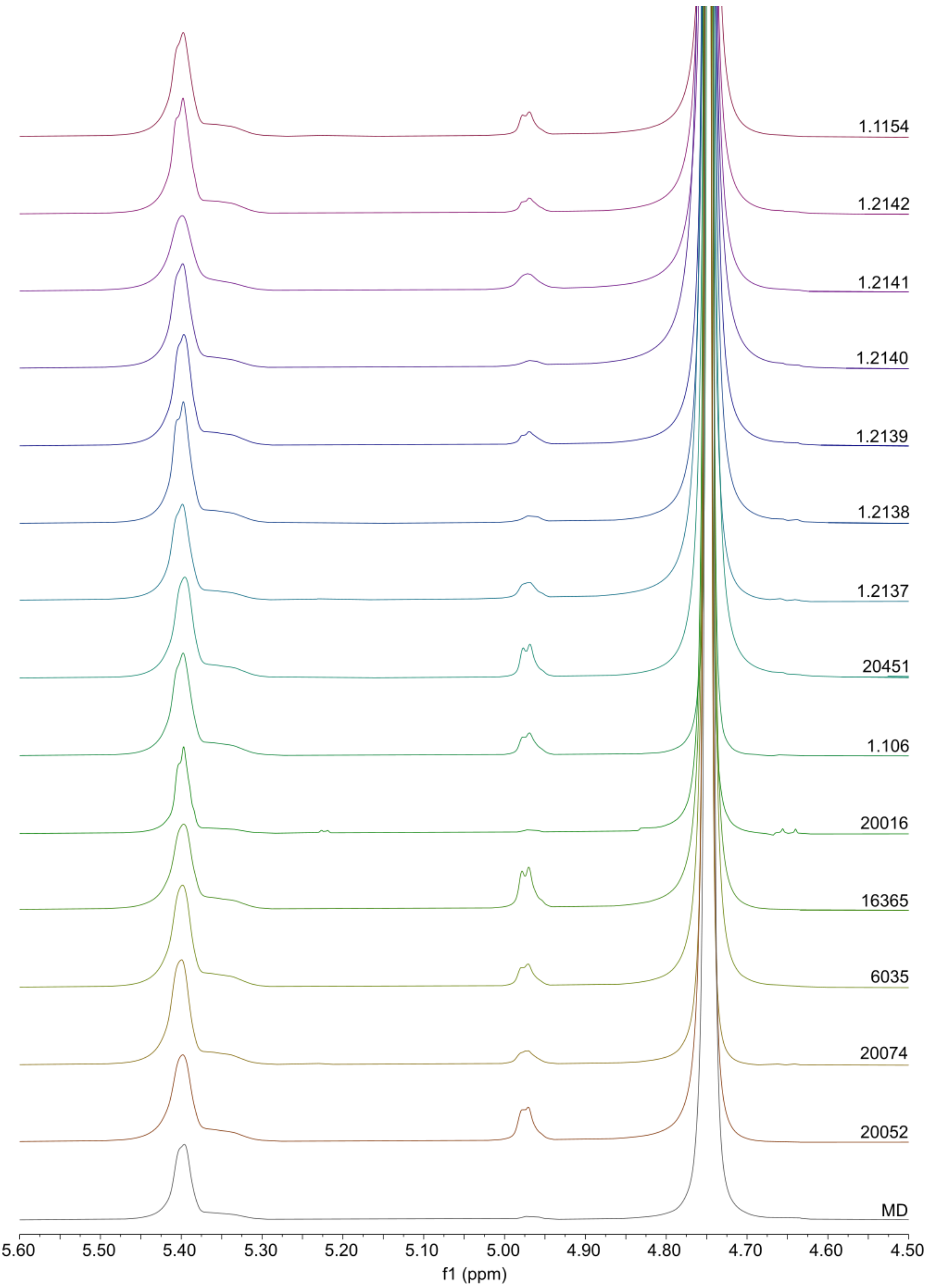

Figure S5. ^{1}H NMR spectra of maltodextrin with a dextrose equivalent of 6.6 (MD) and the isomalto/malto-polysaccharides that were synthesized by different lactic acid bacteria from this maltodextrin. The strains corresponding to the strain numbers are shown in Table 1.

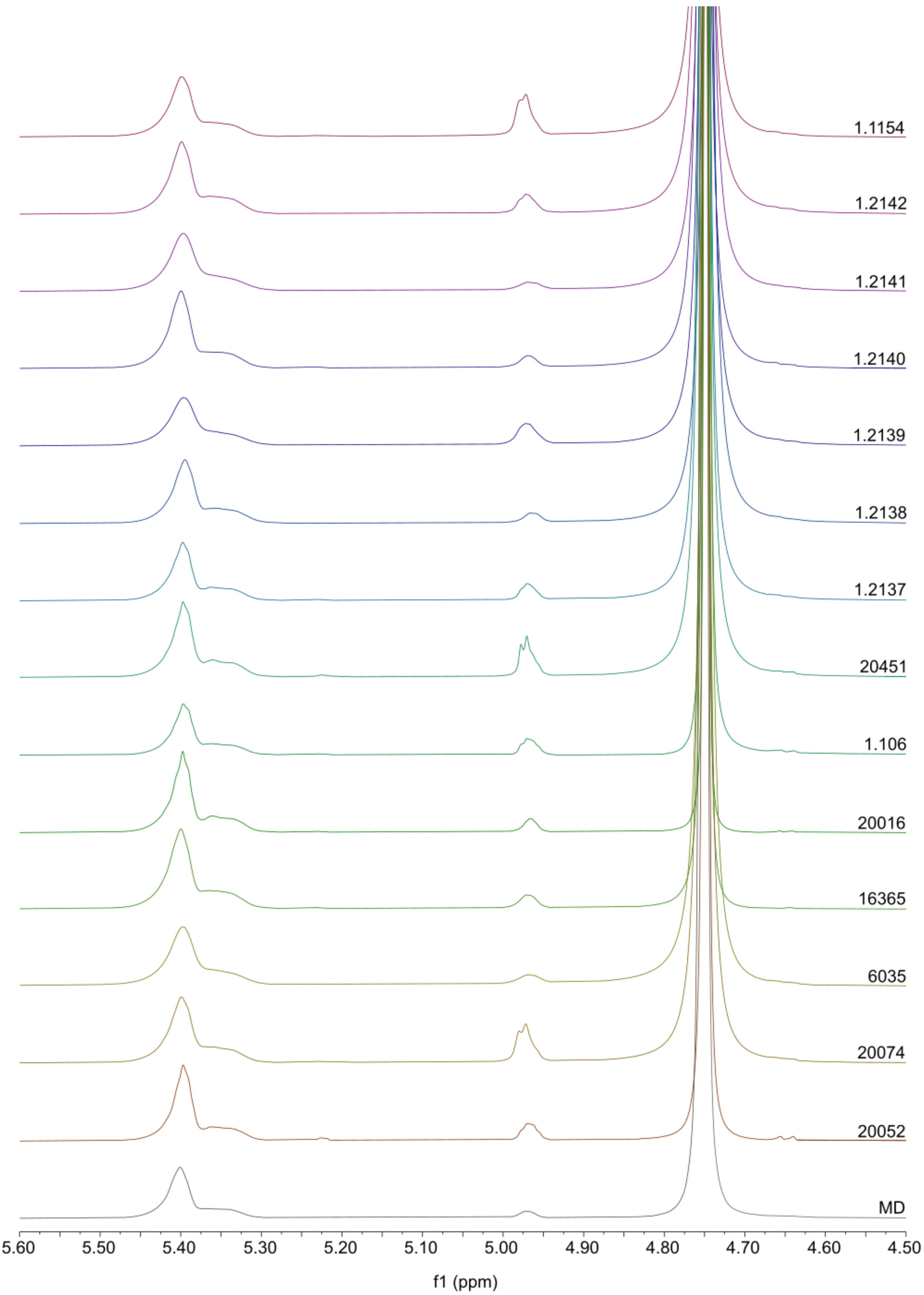

Figure S6. ^{1}H NMR spectra of maltodextrin with a dextrose equivalent of 16.8 (MD) and the isomalto/malto-polysaccharides that were synthesized by different lactic acid bacteria from this maltodextrin. The strains corresponding to the strain numbers are shown in Table 1.

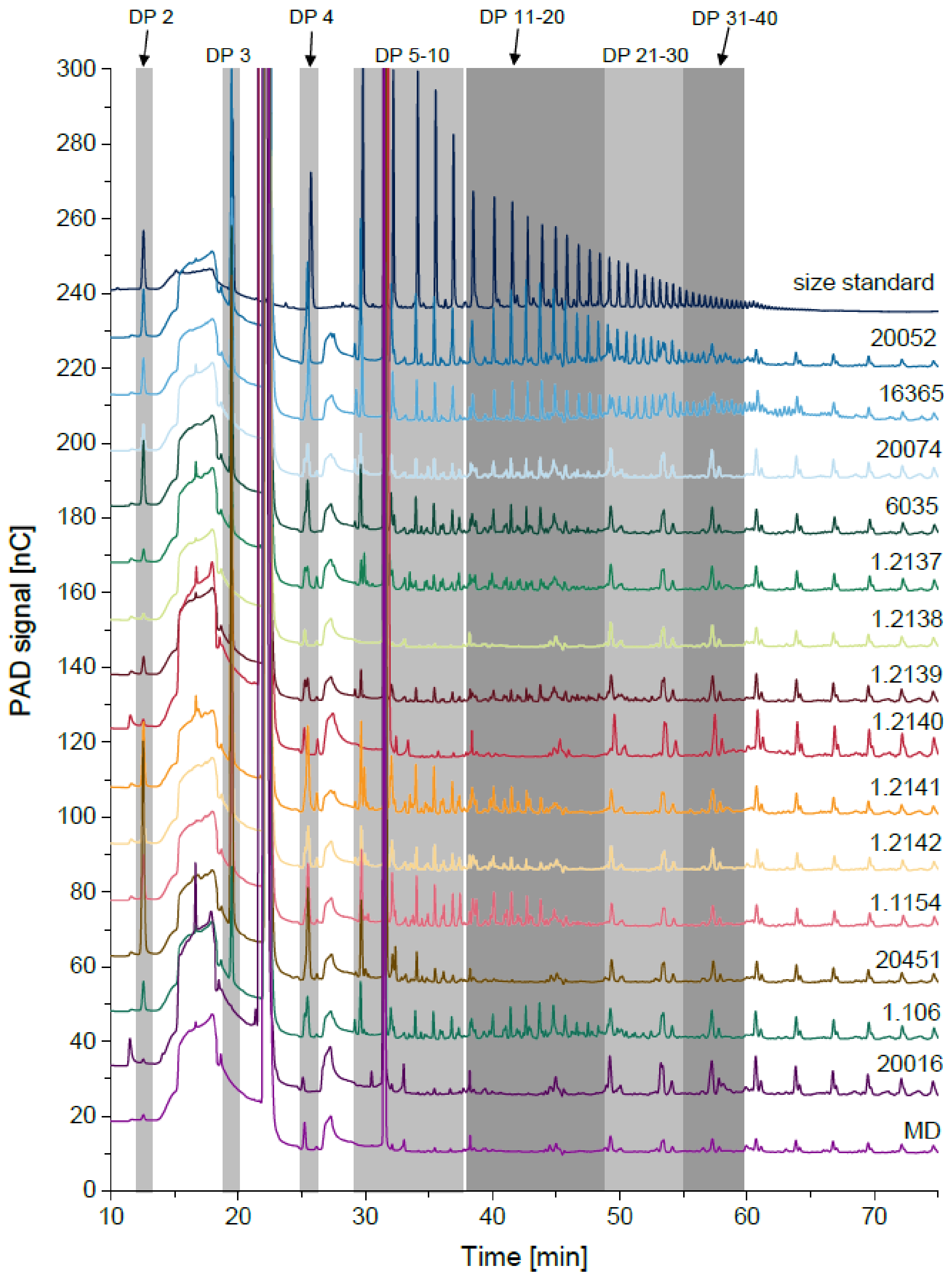

Figure S7: HPAEC-PAD chromatograms of the size standard (partially hydrolyzed linear dextran, dark blue), the hydrolyzed isomalto/malto-polysaccharides (IMMPs) synthesized by different lactic acid bacteria from maltodextrin with a dextrose equivalent of 6.6, and the hydrolyzed maltodextrin (MD) with a dextrose equivalent of 6.6. The degrees of polymerization (DP) of the size standard peaks are marked with grey boxes. The strains corresponding to the strain numbers are shown in Table 1.

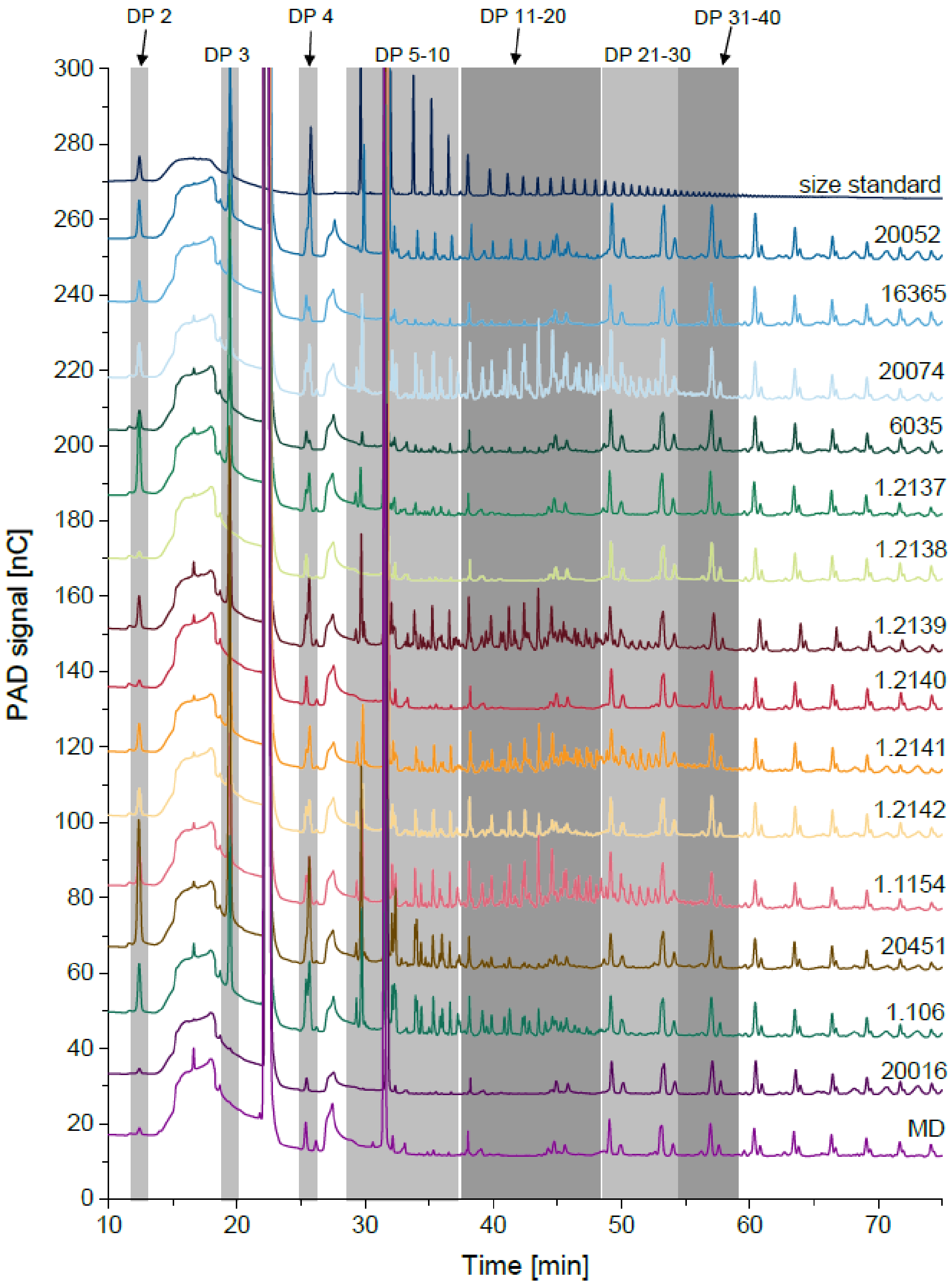

Figure S8: HPAEC-PAD chromatograms of the size standard (partially hydrolyzed linear dextran, dark blue), the hydrolyzed isomalto/malto-polysaccharides (IMMPs) synthesized by different lactic acid bacteria from maltodextrin with a dextrose equivalent of 16.8, and the hydrolyzed maltodextrin (MD) with a dextrose equivalent of 16.8. The degrees of polymerization (DP) of the size standard peaks are marked with grey boxes. The strains corresponding to the strain numbers are shown in Table 1.
